# PICU diaries and parental PTSD symptoms—a pilot randomized controlled trial assessing feasibility and psychological outcomes

**DOI:** 10.3389/fped.2026.1819949

**Published:** 2026-07-21

**Authors:** Franziska Perdereau, Felix Neunhoeffer, Maria Schweizer, Katharina Maier, Ute Wagner, Johannes Nordmeyer, Juliane Engel

**Affiliations:** Department of Pediatric Cardiology, Pulmonology and Intensive Care Medicine, University Hospital Tuebingen, Tuebingen, Germany

**Keywords:** family centered care, ICU diary, PICU (pediatric intensive care unit), pilot study, PTSD, RCT—randomized controlled trial, resilience

## Abstract

**Introduction:**

Parents of children admitted to pediatric intensive care units (PICUs) are at increased risk of post-traumatic stress disorder (PTSD), potentially affecting long-term family well-being. This analysis evaluates the feasibility of a pilot randomized controlled trial (RCT) of PICU diaries, including recruitment, retention, and data collection, while providing insights into intervention implementation. Additionally, selected exploratory findings are reported, including parental trauma-related symptoms at six months post-discharge and the potential influence of gender and resilience on suspected PTSD.

**Methods:**

This pilot RCT was conducted in the PICU of a university hospital. Families were randomized to an ICU diary intervention group (IG) or control group (CG), with stratification by child age. Feasibility outcomes included integration into routine intensive care, family recruitment and retention, and completeness of follow-up. Parents completed questionnaires at admission (T0), PICU discharge (T1), and six months post-discharge (T2). PTSD symptoms were assessed using the German Impact of Event Scale-Revised, while resilience was measured through the Resilience Scale-13.

**Results:**

Of 149 eligible families, 104 (69.8%) consented to participate, resulting in a final sample of 150 parents. Dropout until PICU discharge (T1) was 4.7%, while retention from T1 to the 6-month follow-up (T2) was 95.5%. In exploratory analyses, 19 (12.7%) parents screened positive for suspected PTSD after 6 months, providing preliminary estimates for future sample size calculations. Prevalence of suspected PTSD was 14.5% (10/69) in IG and 11.1% (9/81) in CG (OR = 1.36; 95% CI: 0.52–3.56). PTSD rates appeared higher in mothers than fathers (18.5% vs. 5.8%; OR = 3.69; 95% CI: 1.13–13.21). Parents who screened positive were more likely to exhibit lower resilience.

**Conclusion:**

This pilot study demonstrates that a RCT of ICU diaries in a PICU setting is feasible, with successful recruitment, high retention, and efficient data collection procedures. The intervention was integrated into routine care without indication of harm. Preliminary findings provide important estimates to inform the design and sample size of a fully powered trial. Future studies should further examine potential subgroup effects, including gender and resilience, as well as the impact of diary interventions at multiple time points following discharge.

## 1 Introduction

A child's admission to a pediatric or neonatal intensive care unit (PICU/NICU) is widely recognized as a highly stressful event for parents, often associated with significant psychological distress (1). A growing body of research shows that this experience can lead to posttraumatic stress symptoms (PTSS) and posttraumatic stress disorder (PTSD), affecting approximately 10%–40% of parents of critically ill children (2, 3). Across multiple studies, mothers showed higher rates of PTSD than fathers, with prevalence estimates ranging from 15% to 48% for mothers and 4.3% to 19% for fathers (3). When data from three studies were pooled, mothers were found to have significantly greater odds of developing PTSD compared to fathers (OR = 2.3; 95% CI: 1.3–4.1; *p* < 0.01) (3). In recent years, there has been an increasing focus on the long-term sequelae of pediatric critical care treatment. The Post-Intensive Care Syndrome (PICS) concept was introduced in 2010 by the Society of Critical Care Medicine with a view to capturing cognitive, physical and psychological sequelae in adult ICU survivors (4). It was later expanded to include pediatric patients and their families (PICS-p), acknowledging the interdependent recovery process between patients and their caregivers (4, 5). Emotional and psychological distress in parents is increasingly recognized as a core component of post-PICU outcomes. ICU diaries have been proposed as a low-cost, family-centered intervention to support emotional processing, enhance communication, and reduce trauma-related symptoms (6, 7). First introduced in Scandinavia in the 1970s and later adapted in Germany (8, 9), ICU diaries are now increasingly used in adult settings. While qualitative studies report high acceptance among families and staff in pediatric environments (10–13), quantitative evidence remains limited, particularly from randomized controlled trials (RCTs). The results of RCTs in adult ICU settings have been inconsistent regarding the impact of ICU diaries on PTSD symptoms in family members. A significant reduction (*p* = 0.03) among family members in an intervention group using ICU diaries was observed in one study (14), while others reported no significant effects (15, 16). Meta-analyses also yielded inconsistent findings: Nydahl et al. reported a significant positive effect on family members (17), whereas three other meta-analyses did not find a significant benefit (18–20). In pediatric settings, only two RCTs have hitherto been published (21, 22). One RCT demonstrated a significant (*p* = 0.04) reduction in PTSD symptoms among parents who used an online diary during their child's stay in a NICU (21), whereas a RCT conducted in a PICU setting found no statistically significant differences between the diary group and the control group (22). Beyond such external interventions, individual psychological resources may play a crucial role in shaping parental outcomes following a PICU stay. Resilience is recognized as the ability to adapt successfully to challenging situations (23). A prospective study found that higher parental resilience had a positive effect on psychopathological outcomes, although this was moderated by perceived stress during the PICU stay (24). Similarly, a prospective observational study reported a negative association between parental resilience and self-reported psychological symptoms, suggesting that resilience may serve as a useful marker of psychological risk following PICU stay (25).

During the implementation of PICU diaries for families in an interdisciplinary pediatric intensive care unit, a pilot study was conducted to support the design of future larger-scale trials aimed at improving family-centered care (FCC) and reducing long-term impairment in patients and their parents. To evaluate the feasibility of including multiple family members in a complex study design, data were collected on parental resilience, health-related quality of life in patients and parents, parental satisfaction with the PICU stay, and post-traumatic stress symptoms in parents and children (26). Accordingly, this article provides methodological insights from this study and highlights its limitations and areas for improvement. It further reports on selected exploratory analyses derived from the broader pilot study, examining the potential effects of PICU diaries on rates of suspected PTSD among parents after PICU discharge, subgroup variation according to parental gender, and potential associations between parental resilience and PTSD outcomes.

## 2 Material and methods

### 2.1 Study design and setting

This work presents findings derived from a larger pilot RCT (26). The parent trial was designed as a single-center, non-blinded, randomized controlled pilot study and was conducted in the 12-bed Pediatric Intensive Care Unit (PICU) of a university hospital between March 2023 and November 2024. The objective of the present analysis was to evaluate the feasibility of randomized controlled trials assessing the effect of PICU diaries on psychological outcomes, particularly symptoms of PTSD in parents of critically ill children. Families in the intervention group (IG) received a PICU diary that was jointly completed by healthcare staff and parents, whereas the control group (CG) received standard PICU care without a comparable diary. Data were collected at three distinct time points: at admission (T0), at PICU discharge (T1), and six months after PICU discharge (T2). Due to the active involvement of healthcare staff and families in the diary intervention, blinding was not feasible. The study was approved by the Ethics Committee of the University of Tuebingen (reference no. 801/2022BO2). Procedures were in accordance with institutional ethical standards as well as the Helsinki Declaration of 1975. Reporting followed the CONSORT 2010 extension for randomized pilot and feasibility trials (27).

### 2.2 Participants, eligibility criteria and randomization

Families were eligible if their child stayed in the PICU for more than 48 h, was aged 0–17 years, and if at least one parent had sufficient language skills and agreed to participate. Cases in which the child died during hospitalization or follow-up were excluded from analysis. Participants were randomized into either the IG or the CG using the REDCap® randomization module. Stratified randomization was applied based on six age categories of the children (0–12 months, 13–24 months, 2–4 years, 5–7 years, 8–12 years, 13–17 years), corresponding to the age groups defined in the PedsQL™ 4.0 Generic Core Scales (28). Participants were randomized 1:1 using simple randomization. One parent or both parents per child were eligible to participate in the study and were assigned to the same study group. In cases of readmission within the six-month period between assessment points T1 and T2, children remained in their originally assigned group, and the intervention or standard care was continued accordingly.

### 2.3 Study procedures

#### 2.3.1 The intervention

The PICU diary intervention was developed by an interdisciplinary study team consisting of physicians, nurses, social care workers, and students. The intervention was based on existing ICU and PICU diary concepts (10, 29). The diary was supplied in A4 format as a ring binder, with refillable, pre-punched pages available on site. It was intended to be jointly completed by parents and healthcare staff throughout the child's PICU stay. All professionals involved in the child's care, including nurses, physicians, physiotherapists, and psychotherapists, were encouraged to contribute entries. The diary was stored in the patient-specific bedside drawer located adjacent to the child's bed, ensuring accessibility to both parents and healthcare professionals. Participating children in the IG were identified by labels at the bedside and participation in the study was documented in the clinical information system ICCA® (IntelliSpace® Critical Care and Anesthesia). Materials for individual design, including writing and coloring supplies were provided. With parental authorization, photographs were taken exclusively for diary documentation, printed for inclusion in the diary, and subsequently deleted from the recording device. No fixed number of diary entries per shift was imposed in order to avoid creating an additional burden for healthcare staff during potentially high-workload shifts. Likewise, no specific instructions regarding the writing style of diary entries were provided. Contributors were, however, encouraged to write in a personal and narrative manner, such as directly addressing the child.

The previously described study team was established prior to the start of the intervention. Beyond designing the PICU diary and the study itself, the team was responsible for recruiting families and serving as points of contact for participating parents and ward staff throughout the study. In order to facilitate implementation and reduce potential barriers, a sample PICU diary and a written manual for diary entries were provided to the clinical staff. Additionally, regular presentations were held by the study team. These measures aimed to increase familiarity with the intervention and support diary engagement.

#### 2.3.2 Baseline (T0, PICU admission)

All patients were screened for eligibility according to predefined inclusion criteria. The study team maintained an Excel-based screening log documenting all potentially eligible families, including whether they had already been approached. This ensured a structured recruitment process and prevented parents from being contacted multiple times. A standardized checklist was additionally used to support consistent and systematic inclusion of eligible parents according to the predefined criteria. Subsequently, parents were informed about the study, and written informed consent was obtained using standardized information and consent forms. The randomization of families into the study groups was conducted on site in the presence of the parents. This procedure was undertaken to enhance transparency by demonstrating that allocation was not influenced by the study team. Following enrollment, parental data were recorded in Excel files and in REDCap®. The baseline questionnaires (T0) were dispatched to parents via email. This approach enabled families to prioritize their child's well-being following the consent process and to complete the questionnaires at a later, more convenient time, either on or off the ward. Families allocated to the IG were provided with the PICU diary immediately following randomization.

#### 2.3.3 T1 (PICU discharge)

At T1, parents received follow-up questionnaires via email, including a survey on satisfaction with the PICU stay, which was not included in the present analysis. Additionally, clinical and sociodemographic data of parents and children at discharge were recorded in REDCap®. At this stage, a number of families remained in the hospital on general wards, while others returned home or were in rehabilitation facilities. Families were asked to complete the questionnaires within two weeks of discharge. The diaries were provided to families at the point of discharge.

#### 2.3.4 T2 (6 months post-discharge)

Time point T2 occurred six months after PICU discharge. Questionnaires were administered via email. Non-responders were contacted by telephone after one week, followed by a reminder via SMS after an additional week. Questionnaire responses indicating clinically relevant concerns were referred to the psychosocial support service of the University Hospital Tuebingen. Families were then contacted and, if desired, offered counseling and referral services.

### 2.4 Feasibility outcome measures

Feasibility outcome measures included recruitment-related outcomes, such as the number of eligible families, the number of parents providing consent, and screening rates. Participant retention was further evaluated through dropout rates and loss to follow-up. Data completeness and missing data were evaluated due to the use of a complete-case approach. Furthermore, implementation-related aspects were addressed, reporting on the feasibility of conducting a randomized study in this sensitive clinical setting, the integration of the intervention into clinical routines, and measures to support healthcare staff participation and parental retention.

### 2.5 Exploratory outcome measures

Resilience was assessed at baseline (T0) using the Resilience Scale-13, a validated 13-item short version of the original RS-25 scale developed by Wagnild and Young (30). Each item is rated on a 7-point Likert scale (1 = strongly disagree to 7 = strongly agree), yielding a total score ranging from 13 to 91, with higher scores indicating greater resilience. Based on established cut-offs, resilience can be categorized as low (13–66), moderate (67–72), or high (73–91) (31).

Posttraumatic stress symptoms were assessed at time point T2 using the German version of the Impact of Event Scale–Revised (IES-R), a validated self-report instrument measuring posttraumatic stress symptoms across the domains of intrusion, avoidance, and hyperarousal. The scale consists of 22 items rated on a 4-point Likert scale, with higher scores indicating greater symptom severity. In line with established recommendations for the German version, a regression-based scoring approach was applied to derive a total score, with values >0 indicating a suspected PTSD diagnosis (32, 33).

### 2.6 Data collection

**Figure 1:**
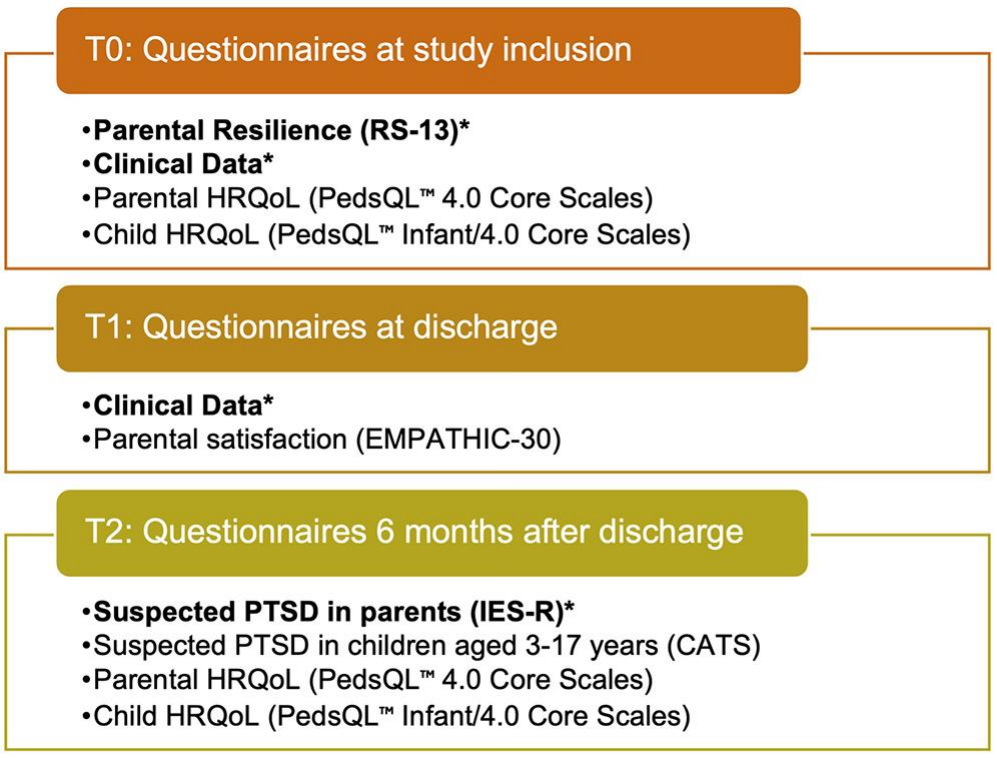
Study questionnaires at time points T0, T1, and T2. RS-13 = Resilience Scale-13; HRQoL = Health related Quality of Life; PedsQL™ = Pediatric Quality of Life Inventory; IES-R = Impact of Event Scale—Revised; CATS = Child and Adolescent Trauma Screen. *This figure illustrates all questionnaires used in the overall study. Measures highlighted in bold were included in the present analysis.

Data were collected at three predefined assessment points: at study inclusion (T0), at discharge from the PICU (T1), and six months after PICU discharge (T2). At baseline (T0), parental resilience was assessed using the validated Resilience Scale-13 (RS-13) (31), and baseline sociodemographic and clinical characteristics of children and parents were recorded. At discharge (T1), clinical characteristics of the child during the PICU stay were documented. At follow-up (T2), symptoms of PTSD in parents were assessed using the German version of the validated Impact of Event Scale-Revised (IES-R) (32). In addition, data on health-related quality of life in parents and children using the PedsQL™ 4.0 Generic Core Scales (T0, T2) (28), parental satisfaction with the PICU stay using the EMPATHIC-30 (T1) (34), and post-traumatic stress symptoms in children over three years of age using the Child and Adolescent Trauma Screen (CATS) (T2) (35) were collected as part of the overall study but were not included in the present analysis. An overview of all questionnaires used in the parent study is provided in Figure 1. Questionnaires were administered electronically via REDCap®. Sociodemographic and clinical data at T0 and T1 were obtained from hospital records (IntelliSpace® Critical Care and Anesthesia system).

### 2.7 Sample size and statistical analysis

The estimation of the required sample size for this study was challenging due to the substantial variability in reported incidences of PTSD among parents of critically ill children. A systematic review reported PTSD prevalence rates ranging from 10% to 42%, and subclinical PTSD symptoms in 18% to 62% of parents following PICU discharge (2). The target sample size was set at 100 families for feasibility testing and exploratory analyses. A sample size consideration regarding parental PTSD symptoms was conducted to provide guidance for future larger-scale studies. Analyses were descriptive and exploratory in nature, with effect estimates and 95% confidence intervals reported where appropriate. Analyses were conducted using a complete-case approach. Participants lost to follow-up were excluded from analyses. Statistical consultation was provided by the Centre for Pediatric Clinical Studies (CPCS), Tuebingen.

## 3 Results

### 3.1 Participant characteristics

A total of 150 parents of 91 children were included in the study, including 69 parents in the intervention group (IG) and 81 parents in the control group (CG). Of the participating parents, 81 (54.0%) were mothers and 69 (46.0%) were fathers. Median PICU length of stay was 10 days (IQR: 7–17) in the CG and 9 days (IQR: 6–14) in the IG. The proportion of male children was 53.1% in the CG and 64.3% in the IG. Baseline characteristics were generally comparable between groups (Tables 1, 2).

**Table 1:** Baseline characteristics of the children at baseline (T0).

Children	Control group	Intervention group
	(*n* = 49)	(*n* = 42)
Gender		
Male	26 (53.1%)	27 (64.3%)
Age of children, m	19.4 [0–187]	24.5 [0–191]
Age group		
0–12 months	35 (71.4%)	28 (66.7%)
13–24 months	5 (10.2%)	3 (7.1%)
2–7 years	7 (14.3%)	7 (16.7%)
≥8 years	2 (4.1%)	4 (9.5%)
Previous PICU stays		
0 stays	26 (53.1%)	26 (61.9%)
1–2 stays	20 (40.8%)	15 (35.7%)
≥3 stays	3 (6.1%)	1 (2.4%)
Current PICU stay		
Length of stay, d	10 (7–17)	9 (6–14)
Readmission within 6 months	17 (34.7%)	10 (23.8%)
Sibling		
Yes	34 (69.4%)	27 (64.3%)
No	15 (30.6%)	15 (35.7%)
Type of admission to the PICU		
Medical	20 (40.8%)	17 (40.5%)
Surgical (planned)	29 (59.2%)	16 (38.1%)
Surgical (unplanned)	—	9 (21.4%)
Occurrence of delirium*, d		
0	29 (59.2%)	24 (57.1%)
1–2	14 (28.6%)	12 (28.6%)
≥3	6 (12.3%)	6 (14.2%)
Invasive mechanical ventilation		
Yes	45 (91.8%)	38 (90.5%)
No	4 (8.2%)	4 (9.5%)
Length of mechanical ventilation, d	6 (3–10.5)	4.5 (2–11)

Number (percentage); Median (IQR); mean [range]; *n* = number of cases; m = in months; d = in days. *Delirium was measured using the SOS-PD Scores.

**Table 2:** Baseline characteristics of the parents at baseline (T0).

Parents	Control group	Intervention group
	(*n* = 81)	(*n* = 69)
Fathers	36 (44,4%)	33 (47,8%)
Mothers	45 (55,6%)	36 (52,2%)
Age of parents, y	36 (32–38)	35 (30.5–39)
Resilience*	73.67 ± 9.3	71.49 ± 8.9

Number (percentage); Median (IQR); Mean ± standard deviation; *n* = number of cases; y = in years. *Resilience was measured using the RS-13.

### 3.2 Feasibility findings

Of 149 eligible families, 104 (69.8%) consented to participate, comprising 170 parents. The final analytic sample included 150 parents. Exclusions occurred due to incomplete questionnaire data or withdrawal of consent (8 parents), death of the child (8 parents), technical issues during data collection (2 parents), or a PICU stay of less than 48 h (2 parents). Among parents enrolled at PICU discharge (T1), retention at the 6-month follow-up (T2) was 95.5%. The dropout rate due to withdrawal of consent until T1 (PICU discharge) was 4.7%. Participant flow is illustrated in the CONSORT flow diagram (Supplementary Appendix 1). Based on observations during study implementation, the PICU diary intervention was well accepted by clinical staff. Variability in the frequency and extent of entries was noted among both parents and healthcare professionals.

### 3.3 Exploratory clinical findings

#### 3.3.1 Suspected PTSD in parents

**Figure 2:**
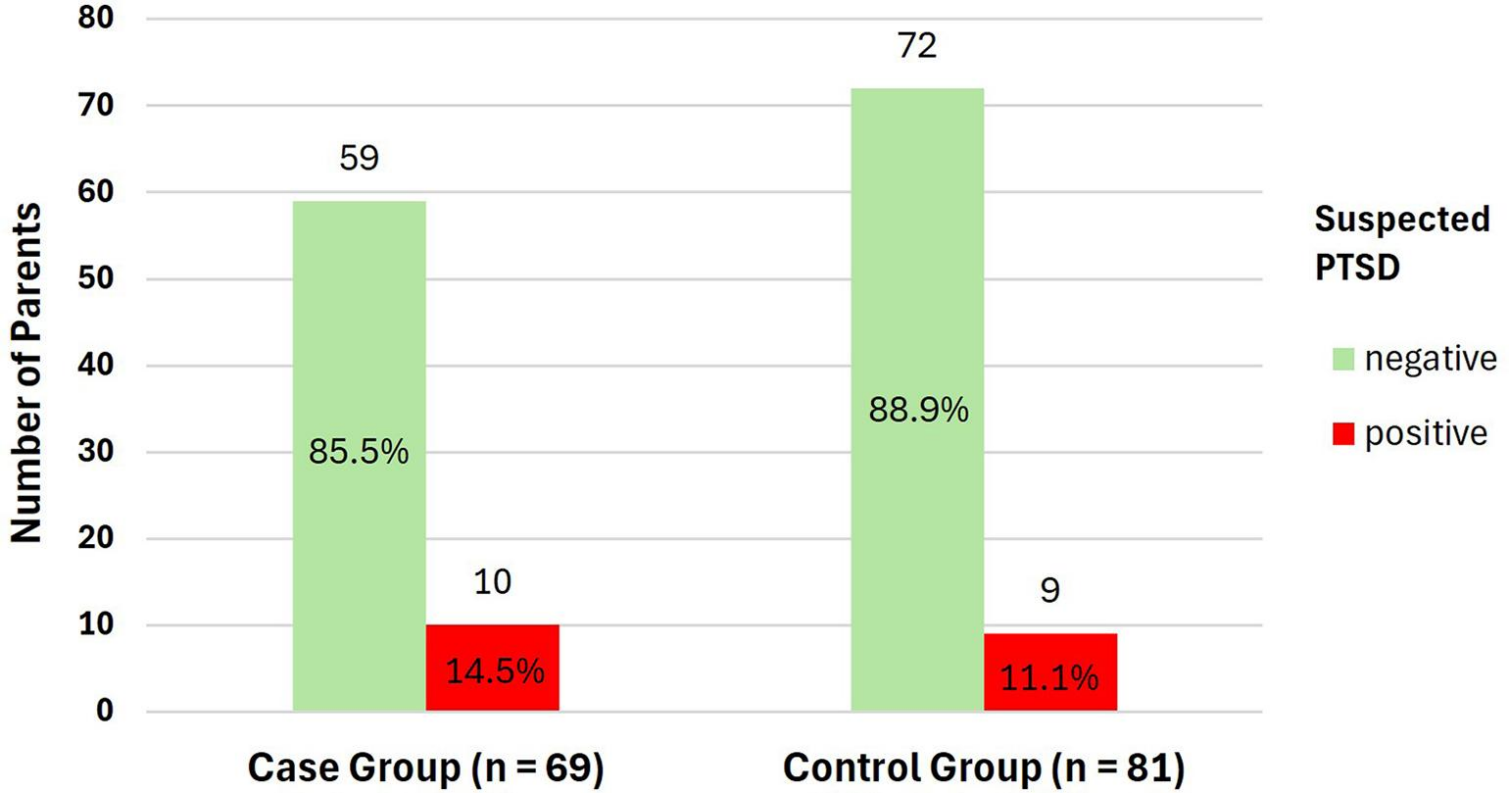
Suspected PTSD in intervention and control groups. *n* = number of cases.

Six months after PICU discharge (T2), 19 parents (12.7%) screened positive for suspected PTSD as assessed using the German version of the IES-R. As illustrated in Figure 2, in the intervention group 14.5% parents (10/69) screened positive for suspected PTSD, compared to 11.1% parents (9/81) in the control group (OR = 1.36; 95% CI: 0.52–3.56). A *post hoc* sample size calculation using NQuery® indicated that approximately 1,600 parents per group would have been required to detect the observed difference with a significance level of 0.05 and a power of 80%.

#### 3.3.2 Suspected PTSD in mothers and fathers

Table 3 presents the distribution of suspected PTSD cases among mothers and fathers. Among the 81 mothers included in the study, 15 (18.5%) screened positive for suspected PTSD, whereas only 4 out of 69 fathers (5.8%) screened positive (OR = 3.69; 95% CI: 1.13–13.21).

**Table 3:** Suspected PTSD among Mothers and Fathers (T2)

**Parents**	**Mothers** **(n = 81)**	**Fathers** **(n = 69)**	**Total** **(n = 150)**	***Odds ratio*** [***95% CI***]
Suspected PTSD* Positive	15 (18.5%)	4 (5.8%)	19 (12.7%)	3.69 [1.13–13.21]
Suspected PTSD* Negative	66 (81.5%)	65 (94.2%)	131 (87.3%)	

Note: Number (percentage), * Suspected PTSD was measured using the German IES-R

#### 3.3.3 Parental resilience and PTSD symptoms

**Figure 3:**
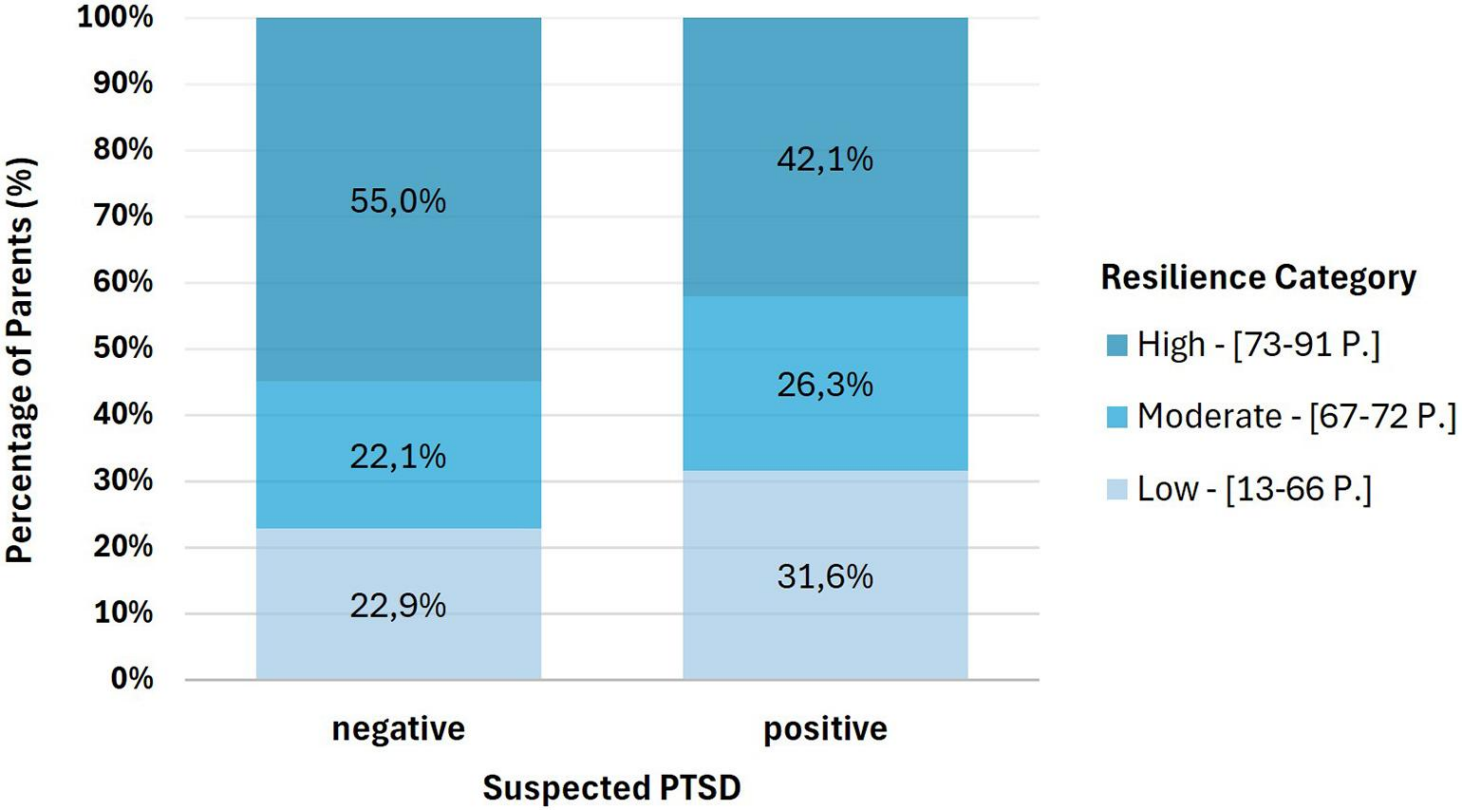
Parental resilience and suspected PTSD. P. = points.

Figure 3 shows the distribution of resilience levels among parents with and without suspected PTSD. Among parents without suspected PTSD, 22.9% (*n* = 30) had low resilience, 22.1% (*n* = 29) moderate resilience, and 55.0% (*n* = 72) high resilience. Among parents with suspected PTSD, 31.6% (*n* = 6) showed low resilience, 26.3% (*n* = 5) moderate resilience, and 42.1% (*n* = 8) high resilience. Low resilience was observed more frequently among parents with suspected PTSD, while high resilience was less common in this group (Cramér's *V* = 0.088). Baseline resilience scores did not differ between groups (Table 2; IG: *M* = 71.49; CG: *M* = 73.67), with a mean difference of −2.18 (95% CI: −0.77–5.12).

## 4 Discussion

### 4.1 Feasibility findings and future directions

The present pilot study provides important insights into the methodological feasibility of conducting larger trials in the PICU setting. The low dropout rate due to withdrawal of consent (4.7%) and the 95.5% retention rate from PICU discharge (T1) to the 6-month follow-up assessment (T2) indicate sustained parental engagement over time and support the successful conduct of longitudinal quantitative research in this clinical context. The applied approach appeared to reduce participation barriers and to facilitate acceptance of data collection procedures, providing important guidance for future trials. In such a demanding setting, study designs must be context-sensitive and family-centered, ensuring that participation does not impose additional burden on families. The well-being of both the child and their parents should remain the foremost priority.

Several methodological and implementation-related aspects emerged during the conduct of the study that may be relevant for future larger-scale studies: As the PICU diary was newly introduced in the PICU setting, the intervention required time to be integrated into existing clinical routines. Initially, uncertainty and hesitancy toward diary writing among staff had to be addressed, particularly as diary engagement required additional time and prioritization within an already demanding clinical environment. To support implementation, the interdisciplinary study team provided regular short briefings during morning meetings as well as targeted presentations. Furthermore, example PICU diaries and written manuals helped familiarize staff with diary use. At the same time, the acute phase of critical illness represents a particularly demanding period for parents, who are often emotionally burdened and primarily focused on their child's condition (36). This may affect both recruitment and data completeness. In this context, the use of electronic questionnaires proved to be both feasible and well accepted by parents, particularly as it allowed them to complete assessments at a convenient time and outside the often stressful clinical environment. Additionally, structuring the diary as a ring binder with removable pages that could be added upon request appeared beneficial. This flexible format enabled both parents and healthcare staff to contribute to the diary independently whenever opportunities arose. It also allowed parents to complete diary pages outside the PICU setting in a quieter environment when desired. Notably, the involvement of a dedicated study coordinator, who was personally known to participating families, emerged as a key facilitating factor. In line with previous research highlighting the important role of study coordinators in participant recruitment and retention, this may have contributed to higher study participation and improved follow-up rates (37).

The findings further suggest the following methodological considerations. In the present study, randomization was stratified according to patient age, but not according to estimated length of PICU stay or severity of illness. Although these characteristics were ultimately well balanced between groups, future trials may benefit from additional stratification according to these variables to more reliably ensure group comparability, as they may influence parental psychological outcomes and intervention effects. Moreover, PTSD was assessed using the Impact of Event Scale–Revised (IES-R) and analyzed as a binary outcome, primarily to allow comparability with the only available quantitative study on ICU diaries in a pediatric setting at the time (21). Future studies may benefit from assessing PTSD symptoms continuously using instruments such as the PTSS-14 to better capture symptom severity, including subclinical changes and smaller intervention effects that may not be reflected by dichotomous classifications. Furthermore, variability in both parental and staff contributions to the PICU diary was observed. As the present study employed a purely quantitative approach, these differences were not explored in greater detail. Given that the extent and nature of diary engagement may influence intervention effectiveness, future studies may benefit from mixed-methods designs integrating both quantitative and qualitative approaches.

### 4.2 Exploratory clinical findings

#### 4.2.1 Suspected PTSD in parents

The present pilot study found no substantial difference in rates of suspected PTSD six months after PICU discharge between parents who received a PICU diary and those who received standard care. These results are in line with previous randomized studies in adult populations, which also reported comparable outcomes between intervention and control groups (15, 16). No relevant confounders, such as differences in parental resilience, child age, or unplanned admissions, were found to account for group differences (Tables 1, 2). The *post-hoc* sample size calculation further indicated that substantially larger sample sizes would be required in future studies to detect potential group differences with sufficient statistical power. Observed PTSD rates in both groups fall within the lower range reported in systematic reviews and meta-analyses (2, 3). This may reflect the high standard of psychosocial care in the study setting, where the diary intervention was implemented as part of a comprehensive family-centered care approach.

A previous RCT by Li et al. in a neonatal intensive care unit (NICU) observed significantly lower parental PTSD symptom levels one month after discharge following the use of ICU diaries (21). In a more recent pilot RCT conducted in a PICU setting (2026), parents in the diary group had lower rates of suspected PTSD one month after discharge compared to controls, although psychological symptoms decreased over time, attenuating the between-group differences observed at earlier follow-up assessments (22). While differences between NICU and PICU settings may have influenced these outcomes (36, 38), the findings suggest that the beneficial effects of PICU diaries on parental PTSD-related symptoms may become less pronounced over time. Consistent with this interpretation, no substantial differences in rates of suspected PTSD between groups were observed six months after discharge in the present study.

PICU diaries appear to serve multiple, clinically relevant functions for parents. First, the diaries allow parents to maintain emotional connection with their child during periods of separation, particularly when sedation limits direct interaction (39). Moreover, they may help reduce informational discontinuity by documenting events occurring in the parents' absence, with entries by healthcare professionals bridging these gaps and facilitating retrospective understanding of the clinical course (12). Crucially, diaries may also operate as a form of therapeutic writing. By enabling parents to externalize and structure their internal experiences, diary use may facilitate emotional processing and provide relief during the acute phase of critical illness (11, 39). In contrast, potential long-term functions, such as reflecting on the experience with the help of the PICU diaries after discharge, may be less influential than these immediate effects. Taken together, the effectiveness of PICU diary use appears to depend on temporal and contextual factors. Based on findings from numerous previous qualitative studies, keeping an PICU diary does not appear to have any harmful effects, supporting its continued use as a potentially supportive tool for families (12, 40, 41). Further investigation of the functions of PICU diaries, particularly regarding short-term psychological outcomes, is warranted.

#### 4.2.2 Suspected PTSD in mothers and fathers

Our exploratory analysis further revealed gender differences, with mothers more likely to screen positive for suspected PTSD than fathers. There were no relevant differences in the baseline characteristics between mothers and fathers that could explain the observed outcome. It is consistent with existing research and may be explained by gender-specific differences in coping mechanisms (3, 42). Overall, the results highlight the importance of targeted support for mothers, who may represent a group at increased risk.

#### 4.2.3 Parental resilience and PTSD symptoms

An inverse relationship was observed between parental resilience levels and the likelihood of suspected PTSD, independent of intervention or control group status. Consistent with previous research demonstrating a low-to-moderate negative correlation between resilience and PTSD symptom severity (43), these findings suggest that resilience may represent a protective factor against PTSD in this population and may have potential value in identifying parents at increased risk for psychological distress. This association should be explored further in larger studies.

In summary, the exploratory analyses may help inform the selection of clinically relevant outcomes and the design of future studies. However, these observations should be considered hypothesis-generating and require confirmation in adequately powered studies. Findings across studies, including the present study, suggest that short-term effects of PICU diaries on parental PTSD-related symptoms may be more pronounced than long-term effects (21, 22). Future research could therefore examine the effects of diary interventions during the acute phase and at multiple time points following discharge. The potential relationship between parental resilience and PTSD also warrants further investigation, particularly with regard to the use of resilience as a potential screening tool.

### 4.3 Limitations

This pilot study has several limitations. Baseline differences in disease severity between intervention and control groups cannot be entirely ruled out and may have influenced outcomes. Therefore, future studies should consider stratified randomization based on age and disease severity (e.g., PRISM). Moreover, blinding of participants and study personnel was not feasible due to the nature of the intervention. This may have introduced response bias, as parents in the PICU diary group were aware that they were receiving an additional intervention and may therefore have felt inclined to report better outcomes than those in the control group. The complete-case approach may also have introduced bias due to the exclusion of participants with missing data. In addition, implementation fidelity may have varied, as the completeness and quality of diary entries depended on the availability and engagement of both healthcare staff and parents, potentially attenuating the effect of the intervention.

## 5 Conclusion

This pilot study demonstrates that conducting quantitative research, including a randomized controlled trial (RCT) of ICU diaries in a PICU setting, is feasible. Recruitment and data collection procedures were successfully carried out, with a 95.5% retention rate between PICU discharge (T1) and 6-month follow-up (T2), indicating sustained parental engagement. The intervention could be implemented within routine intensive care without any indication of harm, and provides valuable preliminary estimates to inform the design and sample size of a fully powered multicenter trial. While PICU diaries were not associated with lower rates of suspected PTSD among parents six months after PICU discharge, exploratory analyses indicated potential subgroup differences. Specifically, suspected PTSD was more frequently observed among mothers than fathers. Additionally, lower parental resilience appeared to be associated with increased PTSD risk. Future research using larger, multicenter, and mixed-methods designs is warranted to further investigate the potential effects of PICU diaries on parental psychological outcomes. Particular attention should be given to subgroup variation (e.g., gender) and the temporal dynamics of diary effects, which may be more pronounced during the early post-discharge period. Further research is also needed to clarify the role of resilience and its potential utility for targeted screening and early psychosocial support for parents at increased risk.

## Data Availability

The datasets presented in this article are not readily available because of the sensitive nature of patient data and data protection regulations, but are available from the corresponding author on reasonable request and with permission of the responsible ethics committee. Requests to access the datasets should be directed to juliane.engel@med.uni-tuebingen.de.

## Supplementary material

Supplementary Appendix 1 CONSORT flow diagram of study participants.
