# Supplementary material for: PICU diaries and parental PTSD symptoms—a pilot randomized controlled trial assessing feasibility and psychological outcomes
Source: Front Pediatr. 2026 Jul 21;14:1819949. doi: 10.3389/fped.2026.1819949 (PMC13433751; doi:10.3389/fped.2026.1819949)
Supplement: Supplementary file 1 [file Datasheet1.pdf]

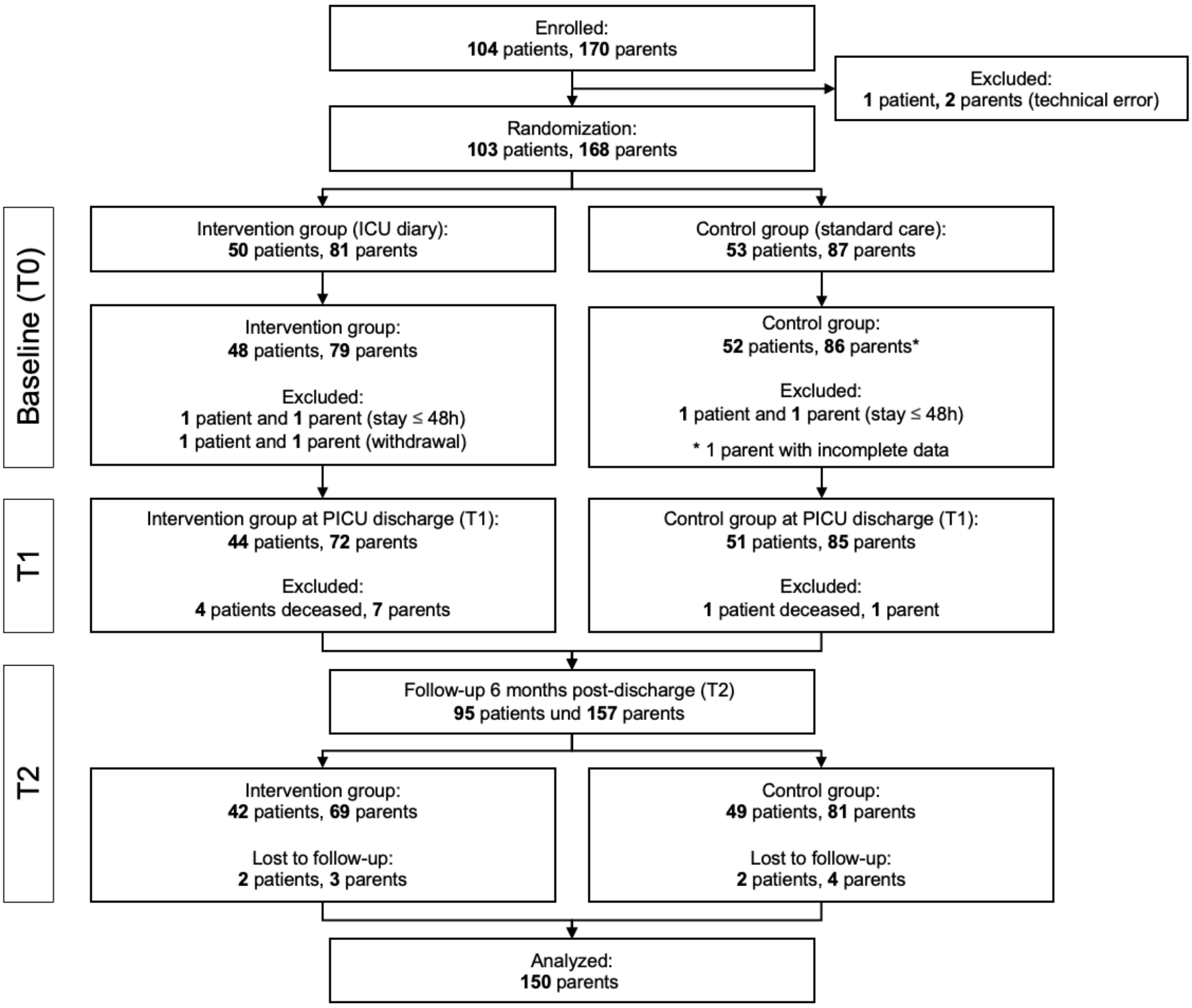

Appendix 1: CONSORT flow diagram of study participants

Note: T0 = baseline (study enrollment); T1 = PICU discharge; T2 = 6 months post-discharge
